# Meeting Report

**DOI:** 10.1155/S1463924684000213

**Published:** 1984


### Meeting Report

### HPLC

The University of Edinburgh's Liquid Chromatography Unit and Hewlett-Packard organized a symposium on aspects of high-performance liquid chromatography column thermodynamics, kinetics and geometry towards the end of 1983 (the column today commands the most attention in HPLC instrumentation). The meeting was held at Cambridge University and there were around 200 delegates from all over the world.

Professor H. Engelhardt (*Saarbruecken University*) began by showing how the selectivity of the stationary phase can be improved by adding small quantities of particular functional groups, although coverage of the silica-gel surface is not uniform and conditions are therefore not easily reproducible, especially on organic molecules. Non-polar eluent is less affected by small changes in concentration and therefore offers better selectivity and reproducibility.

A partial solution to the reproducibility problem was proposed by Dr R. Smith (*Loughborough University*) who has developed a procedure to quantify retention properties and column selectivity by the retention indices of standard compounds. These are chosen to give maximum discrimination between different systems without duplication, allowing the retention properties of different columns to be compared. J. Bruno (*Waters Associates*) then showed how carbon levels available at the surface monolayer affect reverse-phase packing materials and thus retention times. A number of examples were given to demonstrate how carbon loadings correlate with mean particle sizes.

Professor J. Knox (*Edinburgh University*) discussed the thermodynamic aspects of exclusion chromatography, and described a computer model to predict quantitatively the degree of exclusion obtained with the irregular pore geometries of column-packing materials. By suitable inversion it is possible to determine the approximate pore-size distribution of a material from its calibration curve. Then N. R. Herbert (*Shandon Southern*) described recent investigations of a variety of wide-pore materials, including their surface morphologies and size-exclusion characteristics; he presented a series of graphs plotting pore size against pore volume, surface area and distribution.

To conclude the first day, Professor E. Bayer (*Tuebingen University*) moved from the physics to the chemistry of stationary phases, describing the investigation of chemical species on the surface of bonded phases using cross-polarization magic angle spinning carbon-13 NMR. This method overcomes dipole-dipole interactions and reveals conformational differences as well as the different species.

Automatic optimization of the conditions for HPLC separations is difficult as there are so many variables, and the first session on the second day therefore provoked lively discussion, particularly the opening paper from R. P. W. Scott (*Perkin-Elmer Corporation*) on column-design protocols. He has developed a computer program to calculate the optimum column lengths and radius, flow rate and particle size for a given sepration, based on performance criteria, instrument constraints and variable factors pertinent to the analysis. His program also calculates optimum mobile phase velocity.

P. J. Shoemakers (*NV Philips*) has also developed, at the Technische Hogeschool Delft, an optimization routine to establish multi-component mobile-phase mixtures for reverse-phase HPLC separations. The procedure uses a linear interpolation method and is particularly useful when separating all components—the same weight is given to all peaks. An alternative view of optimizing HPLC separations was given by J. C. Berridge (*Pfizer Central Research*)—he has used a sequential Simplex procedure rather than a semi-empirical model. The Simplex, a geometrical model that expands as conditions approach the optimum and contracts when moving in the wrong direction, works by a process of elimination.

Multi-column techniques were discussed in papers from Professors J. F. K. Huber (*Vienna University*) and W. Lindner (*Graz University*). A preliminary separation of complex samples provides fractions that are further separated on a second column, much improving chromatographic performance. Huber described some experiments in which peak compression was used to maintain the separation efficiency in each column, while various switching methods to take the mobile phase from one column to another were shown by both speakers. Lindner also discussed ways of altering the stationary- or mobile-phase conditions between columns to improve selectivity, especially for trace analyses, and compared the relative advantages of on-line and off-line multi-column techniques.

Moving on from this, Professor R. W. Frei (*Amsterdam Free University*) discussed sample preparation using one or more columns for pre-concentration and sample cleaning before transferring to the analytical column for final separation. These pre-columns should be short to allow fast sampling under low pressure with little band broadening. Special sorbents are needed for complex samples.

In the final session, R. Jonker and G. Rozing (*Hewlett-Packard*) outlined the problems facing manufacturers of HPLC columns and instrumentation in providing systems capable of fast and accurate separation at a reasonable cost. The various factors in design strategy were discussed—standard time deviation, flow-rate dynamics, peak density, detector capability and component overlap. Professor J. Kraak (*Amsterdam University*) then described recent advances in liquid-liquid HPLC systems, where the stationary phase is saturated with the mobile phase in which the sample is dissolved. This method offers exact reproducibility and the possibility of adjusting phase ratios, but column preparation is difficult and gradient elution impossible.

The programme ended with a presentation from Professor M. Novotny (*Indiana University*) on capillary micro-columns. Separation impedance reduces with column diameter and microbore columns display high performance, low solvent consumption and great flexibility in the use of detectors. Novotny advocated packed capillary columns despite the difficulties in handling, and proposed methods for preparing them. A novel suggestion involves coating a packed fused silica column with a polymer layer, in which a window is made to provide an on-column detector level.

Finally, Professor Knox led a brief general discussion which covered various problems with column packings.

*Information on the meeting was sent to 'JAC' by Hewlett-Packard. Readers wanting to know more about the HPLC conference should contact the company's Enquiry Section at Eskdale Road, Winnersh, Wokingham, Berkshire RG11 5DZ, UK.*

